# Surface area in the insula was associated with 28-month functional outcome in first-episode psychosis

**DOI:** 10.1038/s41537-021-00186-9

**Published:** 2021-11-29

**Authors:** Shinsuke Koike, Mao Fujioka, Yoshihiro Satomura, Daisuke Koshiyama, Mariko Tada, Eisuke Sakakibara, Naohiro Okada, Yosuke Takano, Norichika Iwashiro, Tatsunobu Natsubori, Yinghan Zhu, Osamu Abe, Kenji Kirihara, Hidenori Yamasue, Kiyoto Kasai

**Affiliations:** 1 University of Tokyo Institute for Diversity & Adaptation of Human Mind (UTIDAHM), Meguro-ku, Tokyo 153-8902 Japan; 2 Center for Evolutionary Cognitive Sciences, Graduate School of Art and Sciences, The University of Tokyo, Meguro-ku, Tokyo 153-8902 Japan; 3 University of Tokyo Center for Integrative Science of Human Behavior (CiSHuB), 3-8-1 Komaba, Meguro-ku, Tokyo 153-8902 Japan; 4 The International Research Center for Neurointelligence (WPI-IRCN), Institutes for Advanced Study (UTIAS), University of Tokyo, 7-3-1 Hongo, Bunkyo-ku, Tokyo 113-8654 Japan; 5 Department of Neuropsychiatry, Graduate School of Medicine, University of Tokyo, Bunkyo-ku, Tokyo 113-8655 Japan; 6 Department of Radiology, Graduate School of Medicine, University of Tokyo, Bunkyo-ku, Tokyo 113-8655 Japan; 7 Disability Services Office, The University of Tokyo, Bunkyo-ku, Tokyo 113-8654 Japan; 8 Department of Psychiatry, Hamamatsu University School of Medicine, Hamamatsu City, 431-3192 Japan

**Keywords:** Schizophrenia, Psychosis

## Abstract

Many studies have tested the relationship between demographic, clinical, and psychobiological measurements and clinical outcomes in ultra-high risk for psychosis (UHR) and first-episode psychosis (FEP). However, no study has investigated the relationship between multi-modal measurements and long-term outcomes for >2 years. Thirty-eight individuals with UHR and 29 patients with FEP were measured using one or more modalities (cognitive battery, electrophysiological response, structural magnetic resonance imaging, and functional near-infrared spectroscopy). We explored the characteristics associated with 13- and 28-month clinical outcomes. In UHR, the cortical surface area in the left orbital part of the inferior frontal gyrus was negatively associated with 13-month disorganized symptoms. In FEP, the cortical surface area in the left insula was positively associated with 28-month global social function. The left inferior frontal gyrus and insula are well-known structural brain characteristics in schizophrenia, and future studies on the pathological mechanism of structural alteration would provide a clearer understanding of the disease.

## Introduction

Schizophrenia mainly manifests in late adolescence and adulthood and continues to affect young people life-long. The outcomes of schizophrenia are heterogeneous, and nearly half of the patients with first-episode psychosis (FEP) achieve symptomatic remission and functional recovery^1,2^. A meta-analysis showed that female sex, higher education, working history, shorter duration of untreated psychosis, milder positive and negative symptoms, and premorbid adjustment, are predictors of a better prognosis in FEP^3^. For individuals at ultra-high risk for psychosis (UHR), which is defined as an earlier clinical stage of the psychosis spectrum with ~25% of the individuals transitioning to psychosis in 2 years^4^. a number of studies have attempted to identify the risk factors for later transition to psychosis (UHR-P)^5^. Furthermore, the predictive factors for clinical outcomes in UHR have become the focus since nearly half of the individuals with UHR without transition to psychosis (UHR-NP) still have a poor prognosis^6,7^. Similar to FEP prognosis studies, female sex, older age, milder subthreshold symptoms, greater cognitive function, and better global function are the predictors for better functional outcome^8^.

To understand the symptomatic and functional outcomes in UHR and FEP more clearly, a number of studies have investigated the relationship between the prognosis and psychobiological characteristics using neuropsychological, neurophysiological, and neuroimaging measurements^2,9–13^. Previous magnetic resonance imaging (MRI) studies in FEP have mostly attempted to reveal the relationships between brain characteristics and treatment response in the short term^10,11^, Gyrification pattern studies in FEP have shown that non-responders to antipsychotic administration at 12 weeks had hypo-gyrification in the left superior and middle frontal gyrus and bilateral insula^14^, and reduced gyrification-based connectivity compared with the responders^15^. For UHR, although many MRI studies have focused on the difference between UHR-P and UHR-NP^9,12,13^, a structural MRI study with a 6-year follow-up showed that people with poor functional outcomes had smaller volume and cortical thickness in the right caudal middle frontal, orbitofrontal, frontal pole, and triangular part of the inferior frontal gyrus (IFG), and the left precuneus and rostral middle frontal gyrus, and smaller subcortical volume in the corpus callosum and nucleus accumbens, compared with those without poor outcomes^16^. A machine learning classification study with a 4-year follow-up revealed that people with poor functional outcomes had reduced cortical surface area in the left superior temporal gyrus and pericalcarine cortex, and the right opercular part of the IFG and cuneus^17^. These results suggest that brain alteration in the prefrontal and temporal cortex could be a factor for long-term prognosis in UHR and FEP.

Although previous studies have attempted to investigate the prognosis using one modality, very few studies have used multi-modal measurements. We recently reported that the deficits in verbal fluency mediated the relationship between the cortical surface area in the right banks of the superior temporal sulcus and 4-month negative symptoms in UHR^18^, suggesting that analyzing the relationship between the characteristics only from one modality and clinical outcomes could preclude an actual relationship. To the best of our knowledge, there has been no study that investigates the relationship of multi-modal measurements with long-term outcomes in UHR and FEP. A multi-modal investigation was proposed for a better understanding of clinical prognosis and the objective markers^12,19,20^.

The Integrative Neuroimaging Studies in Schizophrenia Targeting for Early Intervention and Prevention (IN-STEP) research project was designed as a prospective observational study to explore the pathophysiological features around the onset of psychosis and investigate the possible predictive biomarkers for clinical use since 2008^21^. In this project, we obtained multi-modal psychobiological measurements including a cognitive battery, electrophysiological response during mismatch negativity (MMN), structural MRI, and brain activity using functional near-infrared spectroscopy (fNIRS) for UHR and FEP. For the correlational analyses using future prognosis, we previously reported the relationship of brain activity in the middle frontal gyrus with the later global function score in FEP^22^, duration MMN with later symptomatic remission and frequency MMN with later cognitive function in UHR^23^, and attention/processing speed in UHR-NP and executive function in FEP with 12-month global function^24^. Multisite MRI studies revealed that UHR-P showed higher gyrification in the left occipital lobe compared to UHR-NP^25^. We also reported that the use of a manual tracing method of the IFG subregions that reduced gray matter volume in the triangular part of the IFG was a disease-specific feature of the schizophrenia spectrum (UHR, FEP, and chronic schizophrenia), whereas reduced gray matter in the opercular part was a feature of autism spectrum disorder^26–28^. A multi-modal study showed that the volume in the triangular part of the IFG was associated with fNIRS brain activity in the IFG during a verbal fluency task^29,30^. Now, the data of all modalities at baseline and the clinical follow-up assessments for >2 years are available.

The present study aimed to explore which demographic, clinical, and psychobiological characteristics derived from multi-modal measurements are associated with symptomatic and functional outcomes at the 13-month and 28-month follow-ups in UHR and FEP (Tables 1 and 2). The hypothesis was that biological characteristics related to the prefrontal and temporal cortex would predict the future prognosis. To examine our hypothesis, we aimed to test the relationship between demographic and psychobiological characteristics and future clinical outcomes using a univariate regression model.

**Table 1:** Demographic and clinical characteristics in this study.

	UHR (*n* = 38)	FEP (*n* = 29)	*p* value
**Age**	**21.09 (3.53)**	**23.84 (6.05)**	**0.023**
Female, *n* (%)	20 (52.6)	13 (44.8)	0.70
JART25 IQ	106.40 (9.24)	103.28 (10.74)	0.21
Handedness	89.4 (29.1)	85.8 (29.1)	0.62
Self SES	3.16 (1.34)	3.33 (1.24)	0.61
Parental SES	2.11 (0.73)	2.31 (0.62)	0.25
DUP, median month (range)	NA	2.0 (0.0, 66.5)	
GAF	47.03 (10.28)	41.45 (12.66)	0.051
*PANSS 5 factors*			
Positive symptom	10.89 (2.76)	12.55 (4.40)	0.063
Negative symptom	21.00 (6.58)	22.03 (7.32)	0.55
Disorganized symptom	9.53 (2.82)	10.48 (3.05)	0.19
Excitement symptom	5.87 (2.13)	6.28 (2.07)	0.44
Emotional symptom	11.89 (3.04)	10.66 (3.98)	0.15
*Medication dose, mg*			
**Chlorpromazine**	**82.88 (166.42)**	**403.19 (334.38)**	**<0.001**
**Biperiden**	**0.05 (0.33)**	**1.43 (2.18)**	**<0.001**
Diazepam	5.27 (8.04)	7.59 (8.74)	0.271
**Imipramine**	**18.58 (43.86)**	**0.89 (4.72)**	**0.038**

Mean (SD). Bold shows were significant between the group (*p* < 0.05).

*JART25* the 25-item version of the Japanese Adult Reading Test, *SES* socioeconomic status, *DUP* duration of untreated psychosis, *GAF* the Global Assessment of Functioning; *PANSS* the Positive and Negative Syndrome Scale.

**Table 2:** List of demographic, clinical, and psychobiological characteristics in this study.

Category	Features
*Demographic characteristics*	
	Age
	Sex
	JART25 IQ
	Self SES
	Parental SES
	DUP (only in FEP)
*Clinical characteristics*	
	Symptom severity
	GAF
	PANSS 5 factor
	Positive symptom
	Negative symptom
	Disorganized symptom
	Excitement symptom
	Emotional symptom
	Medication equivalent dose
	Chlorpromazine (antipsychotics)
	Biperiden (antiparkinsonian)
	Diazepam (anxiolytics)
	Imipramine (antidepressant)
	Self-report symptom severity
	CES-D
	WHOQOL-BREF
	Physical domain
	Psychological domain
	Social relationships
	Environment
	General impression
*Psychobiological characteristics*	
	BACS-J
	Verbal memory
	Working memory
	Motor speed
	Verbal fluency
	Attention and Processing speed
	Executive function
	Electrophysiological response
	MMN amplitude in duration deviants
	MMN amplitude in frequency deviants
	Structural MRI
	Cortical surface area (34 regions per hemisphere)*
	Cortical thickness (34 regions per hemisphere)*
	Subcortical volume (7 regions per hemisphere)*
	fNIRS
	Brain activity (8 regions per hemisphere)*
	Total number of words generated during the task
	Stanford Sleepiness Scale during the task

^*^All regions were described in supplementary materials.

*JART25* the 25-item version of the Japanese Adult Reading Test, *SES* socioeconomic status, *DUP* duration of untreated psychosis, *FEP* first-episode psychosis, *GAF* the Global Assessment of Functioning, *PANSS* the Positive and Negative Syndrome Scale, *CES-D* the Center for Epidemiologic Studies Depression Scale, *WHOQOL-BREF* the 26-item brief version of the WHO Quality of Life Scale, *BACS-J* the Brief Assessment of Cognition in Schizophrenia Japanese version, *MMN* mismatch negativity, *MRI* magnetic resonance imaging, *fNIRS* functional near-infrared spectroscopy, *SOFAS* the Social and Occupational Functioning Assessment Scale.

## Results

### Demographic characteristics

The UHR group had greater 13-month emotional factor scores assessed using the Positive and Negative Syndrome Scale (PANSS) compared to the FEP group (*p* = 0.013, Table 3). There was no difference in any other outcomes between the groups. Six individuals with UHR had transitioned to psychosis during the follow-up period; however, there was no difference in any demographic characteristic between UHR-P and UHR-NP, except for the diazepam equivalent dose (*p* = 0.014, 21 individuals with UHR-NP used benzodiazepines, but none of the individuals with UHR-P did).

**Table 3:** Clinical outcomes in the UHR and FEP groups.

	UHR	FEP	*p* value
13-month PANSS 5 factors			
Positive symptom	8.64 (2.83)	8.82 (2.75)	0.81
Negative symptom	17.55 (6.82)	18.73 (6.78)	0.53
Disorganized symptom	8.52 (2.76)	8.23 (3.35)	0.73
Excitement symptom	6.42 (2.48)	5.41 (1.79)	0.10
**Emotional symptom**	**9.76 (3.40)**	**7.59 (2.44)**	**0.013**
13-month SOFAS	59.39 (14.55)	55.27 (15.67)	0.29
28-month GAF symptom subscale	59.03 (16.37)	57.71 (13.42)	0.76
28-month SOFAS	60.59 (17.20)	61.71 (12.76)	0.80

Mean (SD). Bold shows significant between the group (*p* < 0.05).

*SOFAS* the Social and Occupational Functioning Assessment Scale, *PANSS* the Positive and Negative Syndrome Scale, *GAF* the Global Assessment of Functioning.

### The relationship between demographic, clinical, and psychobiological measurements and outcomes

**Fig. 1:**
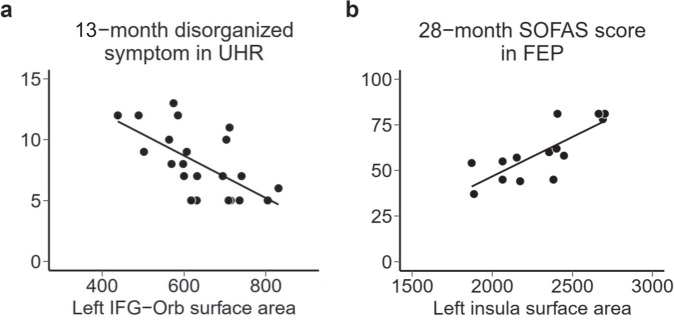
Relationship between psychobiological characteristics and clinical outcomes. **a** The relationship between 13-month disorganized symptom and the surface area in the left orbital part of the inferior frontal gyrus (IFG-Orb) in the UHR group. **b** The relationship between the 28-month Social and Occupational Functioning Assessment Scale (SOFAS) score and the surface area in the left insula in the FEP group.

In the UHR group, the cortical surface area in the left IFG-Orb was negatively associated with the 13-month PANSS disorganized factor score (UHR: *B* = −0.020, *SE* = 0.005, *t* = −4.11, *p* = 0.00054, Fig. 1a; FEP: *B* = −0.006, *SE* = 0.007, *t* = −0.78, *p* = 0.45). In the FEP group, the cortical surface area in the left insula was positively associated with the 28-month Social and Occupational Functioning Assessment Scale (SOFAS) score (UHR: *B* = −0.002, *SE* = 0.016, *t* = 0.14, *p* = 0.89; FEP: *B* = 0.050, *SE* = 0.010, *t* = 4.84, *p* = 0.00052, Fig. 1b).

## Discussion

The present study investigated whether psychobiological characteristics from multi-modal measurements are associated with the symptomatic and functional outcomes at the 13- and 28-month follow-ups for UHR and FEP. The results showed that structural characteristics in the left IFG and insula cortical regions were associated with the symptomatic and functional outcomes. The cortical surface area in the left orbital part of the IFG demonstrated an association with disorganized symptoms in UHR, and in the left insula with global social function in FEP. However, no significant associations were observed for other psychobiological characteristics using a neurocognitive battery, MMN, and fNIRS with long-term outcomes. To the best of our knowledge, this is the first study to identify the relationship between structural characteristics in the left IFG and insula and long-term outcomes in UHR and FEP, using a wide range of demographic, clinical, and psychobiological measurements.

A smaller cortical surface area in the left orbital part of the IFG was associated with severe 13-month disorganized symptoms in the UHR group. We previously reported that individuals in the schizophrenia spectrum (UHR, FEP, and chronic schizophrenia) have more severely reduced gray matter volume in the triangular part of the IFG compared to that in the opercular part^26,28^. In addition, the volume reduction in the right triangular part was associated with severe positive symptoms in UHR^26^, FEP^26^, and chronic schizophrenia^28^. In contrast, individuals with autism spectrum disorders have greater gray matter volume reduction in the opercular part compared that in the triangular part, and the volume is also associated with social communication problems^27^. These studies were conducted using a manual tracing method, and were unable to visualize the orbital part because of technical and image quality reasons. The IFG, especially on the left side, were well-known as the motor speech center, and the orbital part is believed to contribute to the semantic processing of the language. Therefore, disorganized symptoms in UHR may be driven by the structural alteration in this part.

A smaller cortical surface area in the left insula was associated with the 28-month symptomatic and functional outcomes in the FEP group. A longitudinal volumetric MRI study showed that patients with FEP had smaller gray matter volume at baseline and progressive volume reduction in the left insula, and the reduction was correlated with positive and negative symptoms at the 2-year follow-up^31^. Patients with FEP without response to antipsychotic administration at 12 weeks had hypo-gyrification in the bilateral insula compared with the responders^14^. The insula plays a role in interoceptive function, which measures the body and emotional state of the self, and has a connection with a variety of other cortical and subcortical regions^32^. Therefore, the insula function may be related to longer-term outcomes in FEP.

The significant relationships with clinical outcomes were observed in the structural characteristics, which have been well discussed in the schizophrenia spectrum; however, the significant regions were different between the groups. Several reasons could be attributed to the clinical stages and progressive brain pathology. UHR is an earlier clinical stage and most of the participants in this study did non-transition to psychosis in the follow-up, while most of the patients with FEP were diagnosed with schizophrenia. Therefore, the UHR group could demonstrate a more heterogeneous effect on the relationship between the IFG cortical surface area and the 13-month disorganized symptoms. In the FEP group, the progressive brain pathology in the insular cortex could influence the relationship with the 28-month symptomatic and functional outcomes.

Although we previously reported the relationship between functional measurements using fNIRS^22^, MMN^23^, and the BACS^18,24^ and clinical outcomes, there was no correlation with longer outcomes in this study. The longer gap between biological measurements and outcomes could be more related to trait characteristics, such as structural brain images, and repeated functional measurements could be more useful to assess the present condition and short-range prediction^22^.

Several limitations should be mentioned. First, some correlational analyses had small sample sizes, especially for the structural MRI and outcomes in FEP, and future studies with larger sample sizes are warranted to confirm the findings. In this exploratory study, we demonstrated the candidate relationships between structural brain characteristics and future outcomes; we can subsequently need a confirmatory study in the multi-sites obtained on brain images and long-term clinical outcomes in UHR and FEP. Furthermore, future studies should use more sophisticated methods, such as machine learning^16,33,34^. Second, we were unable to obtain detailed symptom severity information, such as that provided by the PANSS, at the 28-month follow-up owing to the naturalistic clinical follow-up. This concern exists especially for young people since they experience more social milestones. In the future, naturalistic longitudinal studies should consider web-based interviews and evaluation methods. Third, we were unable to assess the genetic contribution to the results of this study. Biomarkers from blood collections, such as the polygenetic risk score^35^ may be more useful as a trait marker, and DNA methylation^36,37^, RNA-seq^38^, and blood metabolism^37,39^ may be more useful as state markers.

In conclusion, the present study showed the relationship between structural characteristics in the left IFG and insula, and symptomatic and functional outcomes at the 13- and 28-month follow-ups in UHR and FEP. These regions manifest well-known brain pathology in schizophrenia and their alteration in the early course of the illness could affect long-term outcomes. Future replication studies in multi-sites are warranted for the application of new therapeutic targets and clinical biomarkers for a better prognosis, and for revealing the pathological mechanisms of structural alteration in these regions.

## Methods

### Participants

In the IN-STEP project, we registered 53 individuals with UHR and 37 patients with FEP. Of these, 38 (71.7%) individuals with UHR and 29 (78.4%) patients with FEP who were measured using one or more modalities within 90 days of registration (including cognitive battery, MMN, structural MRI, and fNIRS brain activity) and were assessed at 12-month and/or 28-month follow-ups were included (Table 1). For the remaining 15 individuals with UHR and 8 patients with FEP, no modality data and/or no clinical outcomes were obtained; thus, they were excluded from this study. There was no difference in the demographic characteristics between those included and excluded in this study (Table 4). The availability of demographic and clinical characteristics, psychobiological modalities following preprocessing, and clinical outcomes are shown in Table 5.

**Table 4:** Demographic characteristics between those included and excluded in this study.

	Exclusion	Inclusion	*p* value
*n*	23	67	
*Diagnosis (%)*			0.19
UHR	15 (65.2)	32 (47.8)	
UHR to FEP	0 (0.0)	6 (9.0)	
FEP	8 (34.8)	29 (43.3)	
Age	22.91 (6.08)	22.28 (4.94)	0.62
Female, *n* (%)	8 (34.8)	33 (49.3)	0.34
JART25 IQ	102.68 (10.34)	105.05 (9.96)	0.34
Self SES	3.15 (1.18)	3.23 (1.29)	0.80
Parental SES	2.32 (0.67)	2.19 (0.69)	0.48
DUP	12.54 (17.99)	7.54 (14.27)	0.41
GAF	40.78 (11.11)	44.61 (11.62)	0.17
*PANSS 5 factors*			
Positive symptom	12.96 (3.67)	11.61 (3.63)	0.13
Negative symptom	22.30 (9.69)	21.45 (6.87)	0.65
Disorganized symptom	10.83 (4.57)	9.94 (2.94)	0.29
Excitement symptom	7.17 (3.14)	6.04 (2.10)	0.055
Emotional symptom	9.83 (3.83)	11.36 (3.50)	0.081

Mean (SD). Bold shows significant between the group (*p* < 0.05).

*JART25* the 25-item version of the Japanese Adult Reading Test, *SES* socioeconomic status, *DUP* duration of untreated psychosis, *GAF* the Global Assessment of Functioning, *PANSS* the Positive and Negative Syndrome Scale.

**Table 5:** Number of data available in this study.

	UHR (*n* = 38)	FEP (*n* = 29)
*n*	38 (100.0)	29 (100.0)
JART25 IQ	38 (100.0)	29 (100.0)
Self SES	37 (97.4)	27 (93.1)
Parental SES	38 (100.0)	26 (89.7)
DUP	NA	29 (100.0)
Medication dose	37 (97.4)	28 (96.6)
GAF at baseline	38 (100.0)	29 (100.0)
PANSS at baseline	38 (100.0)	29 (100.0)
CES-D at baseline		
*WHOQOL-BREF at baseline*		
BACS-J	33 (86.8)	28 (96.6)
MMN	29 (76.3)	23 (79.3)
MRI	26 (68.4)	17 (58.6)
fNIRS	34 (89.5)	28 (96.6)
*N of modality*		
1	4 (10.5)	1 (3.4)
2	3 (7.9)	4 (13.8)
3	12 (31.6)	9 (31.0)
4	19 (50.0)	15 (51.7)
13-month follow-up	36 (94.7)	26 (89.7)
28-month follow-up	29 (76.3)	21 (72.4)

*N* (%).

*JART25* the 25-item version of the Japanese Adult Reading Test, *SES* socioeconomic status, *DUP* duration of untreated psychosis, *GAF* the Global Assessment of Functioning, *PANSS* the Positive and Negative Syndrome Scale, *CES-D* the Center for Epidemiologic Studies Depression Scale, *HOQOL-BREF* the 26-item brief version of the WHO Quality of Life Scale, *BACS-J* the Brief Assessment of Cognition in Schizophrenia Japanese version, *MMN* mismatch negativity, *MRI* magnetic resonance imaging, *fNIRS* functional near-infrared spectroscopy.

The participants were recruited from the outpatient and inpatient units of the University of Tokyo Hospital, University of Tokyo Health Service Center, psychiatry clinics, and internet referrals^21^. The inclusion criteria were age 15–40 years for FEP and age 15–30 years for UHR, no antipsychotic medications for psychosis for >16 cumulative weeks, and continuous psychotic symptoms within the past 60 months. All the eligible participants were assessed using the Structured Interview for Prodromal Symptoms (SIPS)^40,41^ by expert psychiatrists, and evaluated using the UHR or psychosis criteria (Supplementary materials). The onset of psychosis, UHR-P condition, was defined according to the SIPS criteria during the 18-month follow-up period, else defined as UHR-NP^23^. Psychosis in the SIPS criteria is the same as psychotic disorders in the Diagnostic and Statistical Manual of Mental Disorders, Fourth Edition^42^. All the diagnostic assessments and clinical severity assessments were checked in the routine meetings in the project, and any concern and inconsistent ratings were discussed and rated by the project psychiatrists and psychologists.

Exclusion criteria were as follows: (1) previous and/or present severe brain injury and/or neurological illness, (2) previous history of electroconvulsive therapy, (3) a premorbid IQ of 70 or less using the 25-item version of the Japanese Adult Reading Test^43,44^, (4) previous and/or present alcohol addiction, (5) previous and/or present continuous substance use, and (6) clear comorbidity with autism spectrum disorders according to the DSM-IV criteria. The detailed inclusion and exclusion criteria of this study are described in the protocol paper^21^.

This study was approved by the ethics committee of the Faculty of Medicine, University of Tokyo (Approval Nos. 397, 629, 630, and 2226), and all the participants, and their caregivers if the participants were under 20 years of age, provided written informed consent to participation in the project and the required measurements following a complete explanation of the experiment.

### Demographic and clinical assessment

Demographic, clinical, and psychobiological characteristics used in this study are listed in Table 2. Handedness was evaluated using the Edinburgh Handedness Inventory^45^. Self and parental socioeconomic status were assessed using the Hollingshead scale^46^. For the FEP group, we defined duration of untreated psychosis as the time period between the onset of the first psychotic symptoms and the initiation of antipsychotic treatment, and this information was obtained from a detailed review of the clinical records or from interviews with patients and their family members using the Nottingham onset schedule^47,48^.

Clinical assessments at baseline were obtained using the Global Assessment of Functioning (GAF)^49,50^ for global symptoms and functions, and the PANSS^51^ for symptom severity by the expert psychiatrists and psychologists in the project. The PANSS scores were categorized into five categories: positive, negative, disorganized, excitement, and emotional symptoms^52^. If the patients were taking any antipsychotic, antiparkinsonian, anxiolytic, and/or antidepressant agents, we calculated the chlorpromazine, biperiden, diazepam, and imipramine equivalent doses, respectively^53^.

The participants reported their subjective depressive symptoms using the Center for Epidemiologic Studies Depression Scale (CES-D)^54^. The CES-D comprises 20 items with a 4-point Likert scale (0 [no symptoms] to 3 [severe]), and the total score was used for subjective depressive symptoms (range 0 to 60). Subjective quality of life (QOL) was obtained using the 26-item brief version of the WHO Quality of Life Scale (WHOQOL-BREF)^55,56^. The WHOQOL-BREF comprises 26 items with a 5-point Likert scale (1 [poor] to 5 [good]). Five factors were calculated in an average score of corresponding items: physical domain, psychological domain, social relationships, environment, and general impression of QOL.

### Psychobiological measurement

We used the Brief Assessment of Cognition in Schizophrenia Japanese version (BACS-J) for the cognitive battery, duration and frequency MMN, T1-weighted structural MRI, and brain activity in the frontotemporal cortical area during a verbal fluency task using fNIRS, for the psychobiological measurement.

The BACS-J^57,58^ measures six cognitive subdomains thought to be affected by schizophrenia: (i) list learning as verbal memory, (ii) digit sequencing task as working memory, (iii) token motor task as motor speed, (iv) category, and letter fluency as verbal fluency, (v) symbol coding as attention and processing speed, and (vi) the Tower of London task as executive function. Trained psychologists conducted the examinations. We used *z* scores for each category standardized by Japanese age-clustered participants^57^.

MMN is a negative component of electrophysiological response elicited by infrequent deviant stimuli occurring within a series of frequent standard stimuli, and it is considered a promising biomarker among other components^59,60^. As in the previous studies^23,61,62^, electroencephalogram was recorded using a 64-channel Geodesic EEG System (Electrical Geodesics Inc., Eugene, OR). We used two types of auditory oddball paradigms using duration and frequency deviant stimuli. Each paradigm had 2000 stimuli consisting of 90% standard tones (1000 Hz, 50 ms) and 10% deviant tones (duration MMN: 1000 Hz, 100 ms; frequency MMN: 1200 Hz, 50 ms). The auditory stimuli were delivered at an 80-dB sound pressure level, 1 ms rise/fall time, and 500 ms stimulus-onset asynchrony. We calculated the duration and frequency of MMN amplitudes as the mean amplitudes from 135 to 205 ms and from 100 to 200 ms post-stimulus, respectively. We used average amplitudes in seven electrodes around the frontocentral electrode as the MMN amplitude (Supplementary materials)^23,61,62^.

Through the project, we used two T1-weighted structural brain image scanning procedures using 3.0-Tesla MRI machines (*n* = 46 and 17, Supplementary materials). Abnormalities and anomalies were checked by a trained neuroradiologist using T1- and T2-weighted images. We excluded images from further analyses if there were abnormalities and anomalies, or substantial head motion and poor quality that could affect preprocessing. After visual inspection, we also excluded one participant because of the failure of preprocessing using FreeSurfer 6.0^63^. Finally, 43 images were used in this study. We extracted cortical and subcortical structural information from preprocessed images and applied 150 variables of the cortical surface area, cortical thickness, and subcortical volume to the analyses (68, 68, and 14 variables, respectively). In the preprocessing phase, we also checked outliers and inspected the segmented region of interest images using the ENIGMA quality control protocol^64^. Since the effect size between scan procedures would affect the analysis, we applied ComBat^65^ to the preprocessed data set to harmonize the data sets (Supplementary materials).

fNIRS is a portable functional neuroimaging instrument used for the convenient and non-invasively measurement of hemoglobin changes over the surface of the cortex^66^. Near-infrared light (650–1000 nm) emitted from a source probe on the human scalp is partially absorbed by the hemoglobin in small vessels (<1 mm), and the remaining light is scattered. A detector probe can subsequently perceive the scattered near-infrared light placed 3 cm away from the source probe in adults^66^.

Brain activity in the prefrontal and anterior temporal cortical surface area during a 160-s block-designed version of the phonological verbal fluency task was obtained using a 52-channel fNIRS instrument (ETG-4000; Hitachi Ltd., Tokyo, Japan) (Supplementary materials)^22,30,66,67^. The obtained activity was checked using automatic rejection software for visible artifacts derived from the body and head movements^30,68^, and two measurements were excluded from further analyses. The location of fNIRS measurements for each channel was estimated using a probabilistic location by a virtual registration from the MRI measurements with an fNIRS probe attachment^69,70^. Relative brain activities according to the task were obtained from the 16 frontotemporal regions^30^. We recorded the total number of correct words during the task period as task performance. Following the measurement, subjective sleepiness during the task was assessed using the Stanford Sleepiness Scale^71^.

### Outcome measures

We set seven outcome measures: 13-month PANSS five factor scores (mean [SD] = 398.7 [57.3] days from registration), and 28-month SOFAS and symptom subscale score of the GAF (mean [SD] = 857.0 [382.4] days). For the 28-month outcomes, we used the SOFAS and symptom subscale of the GAF with more detailed anchor points^49^ in the IN-STEP project procedure^21^. We conducted the long-term clinical outcome assessments during Jan 2018 and Dec 2019.

### Statistical analysis

All analyses were performed using R version 3.6.2 (The R Foundation for Statistical Computing Platform, Vienna, Austria). We explored which of the demographic, clinical, and psychobiological measurements listed in Table 2 were associated with follow-up outcomes using a general linear model. For the variables in structural MRI, the models were controlled for intracranial volume. A difference of uncorrected *p* < 0.001 was considered significant according to the exploratory MRI studies using FreeSurfer features^72–74^ since there was no consensus on the significance threshold set in multi-modal analyses and the sample sizes were too small to tolerate Bonferroni or false discovery rate corrections.

### Reporting summary

Further information on research design is available in the Nature Research Reporting Summary linked to this article.

## Supplementary information

Supplementary Information

Reporting Summary

## Data Availability

All data are available after the approval of the relevant ethical committees. Kindly contact the corresponding author for use of the data set.
