# Supplementary material for: Surface area in the insula was associated with 28-month functional outcome in first-episode psychosis
Source: NPJ Schizophr. 2021 Nov 29;7:56. doi: 10.1038/s41537-021-00186-9 (PMC8630202; doi:10.1038/s41537-021-00186-9)
Supplement: Supplementary file 1 — Supplementary Information [file 41537_2021_186_MOESM1_ESM.pdf]

Surface area in the inferior frontal gyrus and insula was associated with 13- and 28-month outcomes in ultra-high risk and first-episode psychosis

Supplementary materials

Contents

Supplementary Methods 2
Criteria for the UHR group 2
EEG recording and preprocessing in MMN 3
MRI scan procedure 4
Procedure 1 4
Procedure 2 4
List of brain regions 4
ComBat harmonization 5
fNIRS measurement procedure 6
fNIRS instrument 6
Cognitive task 6
fNIRS signal treatment 7
Supplementary References 8

Supplementary Methods

Criteria for the UHR group

The inclusion criteria for the ultra-high risk for psychosis (UHR) group were defined using the Structured Interview for Prodromal Syndromes (SIPS) criteria by assessing the Scale of Prodromal Symptoms (SOPS) [1,2]. The SIPS criteria contain 3 conditions: 1) Attenuated Positive Symptom Syndrome (APSS), 2) Brief Intermittent Psychotic Symptom Syndrome (BIPS), and 3) Genetic Risk and Deterioration Syndrome (GRDS). The criteria of each condition are as follows:

1) APSS

Satisfying all the following items (a–c):

a) The presence of at least one of the following 5 SOPS positive items in the prodromal range (rating of 3–5): P1 (Unusual Thought Content/Delusional Ideas), P2 (Suspiciousness/Persecutory Ideas), P3 (Grandiosity), P4 (Perceptual Abnormalities/Hallucinations), and/or P5 (Disorganized Communication).

b) Symptoms began within the past year or increased by 1 or more points in the SOPS assessment within the past year.

c) Symptoms occurred at least once per week for the last month.

2) BIPS

Satisfying all the following items (a–c):

a) The presence of at least 1 of 5 SOPS positive items in the psychotic range (rating of 6).

b) Symptoms began in the past 3 months.

c) Symptoms occurred at least several minutes per day and at least once per month.

3) GRDS

Satisfying all the following items (a–b):

a) A first degree relative had a history of a psychotic disorder, or criteria for schizotypal personality disorder were met in the patient.

b) The global assessment of functioning (GAF) score dropped at least 30% over the last month compared to 1 year ago.

EEG recording and preprocessing in MMN

Electroencephalogram (EEG) data were recorded using a 64-channel Geodesic EEG System (Electrical Geodesics Inc., Eugene, OR). The electrodes were referenced to the vertex with the impedances being maintained < 50 kΩ. The sampling rate was set at 500 Hz with the analog bandpass filter set at 0.1–100 Hz. The two paradigms were counterbalanced across participants. All stimuli were presented binaurally through earphones while participants sat watching a silent cartoon.

Obtained waveform was analyzed using EEGLAB [3]. EEG signals at each electrode were re-referenced using an average reference and digitally filtered at 0.1–20 Hz. Epochs were extracted from −100 to 500 ms relative to the stimulus onset. Then, the baseline was corrected by subtracting the mean amplitude from −100 to 0 ms. Eyeblink artifacts were extracted using an independent component analysis. Epochs exceeding ± 100 µV at each electrode were excluded. The waveforms evoked by both standard and deviant stimuli were obtained through across-trial averaging. The MMN waveform was obtained as the difference in the average waveforms between the standard and deviant stimuli. We calculated the duration and frequency MMN amplitudes as the mean amplitudes from 135 to 205 ms and from 100 to 200 ms post-stimulus, respectively. We used average amplitudes in seven electrodes around the frontocentral electrode (FCz; Geodesic Sensor Net [GSN] numbers: 3, 4, 5, 8, 9, 55, and 58) as the MMN amplitude.

MRI scan procedure

Procedure 1

T1-weighted images were acquired using a 3.0-Tesla MRI scanner (Signa HDxt; GE Healthcare, Milwaukee, Wisconsin), the standard 8-channel head coil, and three-dimensional Fourier-transform fast-spoiled gradient recalled acquisition with steady state (3D-FSPGR) (repetition time = 6.80 ms, echo time = 1.94 ms, flip angle = 20°, slice thickness = 1.0 mm, field of view = 240 mm, matrix = 256 × 256, number of axial slices = 176).

Procedure 2

T1-weighted images were acquired using a 3.0-Tesla MRI scanner (MR750W; GE Healthcare, Milwaukee, Wisconsin), the standard 32-channel head coil, and 3D-FSPGR (repetition time = 8.50 ms, echo time = Min Full, flip angle = 20°, slice thickness = 1.0 mm, field of view = 240 mm, matrix = 256 × 256, number of axial slices = 176).

List of brain regions

We used the Desikian-Killiany atlas in FreeSurfer for the cortical segmentation listed as follows:

<u>Cortical surface area and thickness (34 regions per hemisphere)</u>

Superior Frontal
Rostral Middle Frontal
Caudal Middle Frontal
Pars Opercularis of the Inferior Frontal
Pars Triangularis of the Inferior Frontal
Pars Orbitalis of the Inferior Frontal
Lateral Orbitofrontal
Medial Orbitofrontal
Precentral
Paracentral
Frontal Pole
Superior Parietal
Inferior Parietal
Supramarginal
Postcentral
Precuneus

Superior Temporal
Middle Temporal
Inferior Temporal
Banks of the Superior Temporal Sulcus
Fusiform
Transverse Temporal
Entorhinal
Temporal Pole
Parahippocampal
Lateral Occipital
Lingual
Cuneus
Pericalcarine
Rostral Anterior (Frontal)
Caudal Anterior (Frontal)
Posterior (Parietal)
Isthmus (Parietal)
Insula

<u>Subcortical volume (7 regions per hemisphere)</u>
Thalamus
Caudate
Putamen
Pallidum
Hippocampus
Amygdala
Nucleus Accumbens

ComBat harmonization

Since data obtained from procedures 1 and 2 were thought to had significant machine- and protocol-derived image difference, we applied ComBat to harmonize the FreeSurfer preprocessed data. For obtaining better harmonized dataset compared to the data used in this study, we used 63 images (Procedure 1 = 46 [UHR = 28, FEP = 18], Procedure 2 = 17 [UHR = 10, FEP = 7]) some of which were not used in this study.

fNIRS measurement procedure

fNIRS instrument

A 52-channel fNIRS instrument (ETG-4000; Hitachi Medical Co., Tokyo, Japan) was used to measure the relative changes in oxygenated hemoglobin concentration that reflect cortical activity. The fNIRS probe attachment was set with 33 probes in a thermo-plastic 3 × 11 shell to cover the bilateral prefrontal and anterior temporal cortices, with the lowest probe line set along the Fp1–Fp2 line defined by the international 10–20 system commonly used in electroencephalography. The location of fNIRS measurements for each channel was estimated using a probabilistic location by a virtual registration from MRI measurements with an fNIRS probe attachment [4,5]. This method provides the probability (p) of a brain region for each channel (ch) within 1 cm of the T3–T4 segment. Using this registration, we estimated brain signals in each brain region of interest (ROI) using the formula:

$$\mathrm{Signal}_{T3T4,ROI} = (\Sigma\, p_{ch} \times \mathrm{Signal}_{ch}) / p_{sum}.$$

The virtual registration for the 52-channel probe covered 12 brain regions in the front-temporal hemisphere using Automated Anatomical Labeling (AAL) [6]. To obtain reliable fNIRS signals in the brain regions within 25–35 cm of the T3–FPz-T4 segment according to the international 10-20 system used in EEG, we used 8 regions per hemisphere for further analyses (superior frontal gyrus, superior frontal medial cortex, middle frontal gyrus, inferior frontal gyrus triangularis, inferior frontal gyrus opercularis, inferior frontal gyrus orbitalis, superior temporal gyrus, and middle temporal gyrus). Missing values for the T3–T4 segment were substituted by the mean length for male and female participants in this study, 31 cm for male participants and 29 cm for female, respectively.

Cognitive task

Participants only needed to sit in a chair in a relaxed state with their eyes open. To minimize motion artifacts, we instructed them to refrain from physical movements such as head motions and strong biting during measurements.

We used a 160-s block-designed phonological verbal fluency task that is well adapted as an activation task during fNIRS measurements [7,8]. The task consists of 30-s pre-task, 60-s task, and 70-s post-task periods. In the 60-s task period, the participant was instructed to say as many words aloud as possible that start with a phonological syllable provided by a computer. The task period was divided into 3 sub-periods, and

the instructed syllables changed every 20 s to avoid silent moments. In the 30-s pre-task and 70-s post-task periods, the participant was instructed to say Japanese vowels aloud repeatedly, to control for task-related motion artifacts and facilitate their removal. This procedure can spatio-temporally measure hemoglobin changes, mainly in the prefrontal cortex and the anterior and superior parts of the temporal cortex, because continuous word generation exercises various cognitive domains involved in verbal storage, verbal working memory, inhibition, and executive control to avoid repetition and inappropriate word use. The number of words generated during the task period was assessed as task performance.

fNIRS signal treatment

Signals for fNIRS oxygenated hemoglobin were acquired for 0.1 s. Then, the signals were focused on task-specific signal changes using a linear fitting between the last 10 s of the pre-task period and the 5 s between the 50- and 55-s time points of the post-task period. Brain activity is defined as relative hemoglobin changes during the task period compared to pre- and post-task periods (mM·mm).

For visible artifacts derived from body and head movements, we used automatic rejection software revised from our previous study [9]. We classified the artifacts contained in fNIRS signals into the following 3 types: flattened signals, Gaussian noise, and motion artifacts. We discarded the entire signal from a channel when it contained artifacts exceeding the predetermined threshold. Therefore, the available channels were different for each participant. If 27 or more channels were rejected, we excluded the measurement itself from the analysis. Finally, fNIRS oxygenated hemoglobin signals were smoothed for 5 s to clear high frequency noises such as heartbeat and small motion artifacts.

Supplementary References

1 Kobayashi, H., Nozaki, S. & Mizuno, M. Reliability of the structured interview for prodromal syndromes Japanese version (SIPS-J). *JPN Bull Soc Psychiat* **15**, 168-174 (2007).

2 Miller, T. J. *et al.* Symptom assessment in schizophrenic prodromal states. *Psychiatr Q* **70**, 273-287 (1999).

3 Delorme, A. & Makeig, S. EEGLAB: an open source toolbox for analysis of single-trial EEG dynamics including independent component analysis. *J Neurosci Methods* **134**, 9-21, doi:10.1016/j.jneumeth.2003.10.009 (2004).

4 Tsuzuki, D. & Dan, I. Spatial registration for functional near-infrared spectroscopy: From channel position on the scalp to cortical location in individual and group analyses. *Neuroimage*, doi:10.1016/j.neuroimage.2013.07.025 (2013).

5 Tsuzuki, D. *et al.* Virtual spatial registration of stand-alone fNIRS data to MNI space. *Neuroimage* **34**, 1506-1518, doi:10.1016/j.neuroimage.2006.10.043 (2007).

6 Tzourio-Mazoyer, N. *et al.* Automated anatomical labeling of activations in SPM using a macroscopic anatomical parcellation of the MNI MRI single-subject brain. *Neuroimage* **15**, 273-289, doi:10.1006/nimg.2001.0978 (2002).

7 Koike, S. *et al.* Application of functional near infrared spectroscopy as supplementary examination for diagnosis of clinical stages of psychosis spectrum. *Psychiatry Clin Neurosci* **71**, 794-806, doi:10.1111/pcn.12551 (2017).

8 Koike, S. *et al.* Association between rostral prefrontal cortical activity and functional outcome in first-episode psychosis: a longitudinal functional near-infrared spectroscopy study. *Schizophr Res* **170**, 304-310, doi:10.1016/j.schres.2016.01.003 (2016).

9 Sakakibara, E. *et al.* Detection of resting state functional connectivity using partial correlation analysis: A study using multi-distance and whole-head probe near-infrared spectroscopy. *Neuroimage* **142**, 590-601, doi:10.1016/j.neuroimage.2016.08.011 (2016).
