# Supplementary material for: Surface area in the insula was associated with 28-month functional outcome in first-episode psychosis
Source: NPJ Schizophr. 2021 Nov 29;7:56. doi: 10.1038/s41537-021-00186-9 (PMC8630202; doi:10.1038/s41537-021-00186-9)
Supplement: Supplementary file 2 — Reporting Summary [file 41537_2021_186_MOESM2_ESM.pdf]

nature portfolio

Corresponding author(s): Shinsuke Koike

Last updated by author(s): Oct 21, 2021

Reporting Summary

Nature Portfolio wishes to improve the reproducibility of the work that we publish. This form provides structure for consistency and transparency in reporting. For further information on Nature Portfolio policies, see our Editorial Policies and the Editorial Policy Checklist.

Statistics

For all statistical analyses, confirm that the following items are present in the figure legend, table legend, main text, or Methods section.

n/a	Confirmed	
☐	☒	The exact sample size (n) for each experimental group/condition, given as a discrete number and unit of measurement
☐	☒	A statement on whether measurements were taken from distinct samples or whether the same sample was measured repeatedly
☐	☒	The statistical test(s) used AND whether they are one- or two-sided<br>*Only common tests should be described solely by name; describe more complex techniques in the Methods section.*
☐	☒	A description of all covariates tested
☐	☒	A description of any assumptions or corrections, such as tests of normality and adjustment for multiple comparisons
☐	☒	A full description of the statistical parameters including central tendency (e.g. means) or other basic estimates (e.g. regression coefficient) AND variation (e.g. standard deviation) or associated estimates of uncertainty (e.g. confidence intervals)
☐	☒	For null hypothesis testing, the test statistic (e.g. F, t, r) with confidence intervals, effect sizes, degrees of freedom and P value noted<br>*Give P values as exact values whenever suitable.*
☒	☐	For Bayesian analysis, information on the choice of priors and Markov chain Monte Carlo settings
☒	☐	For hierarchical and complex designs, identification of the appropriate level for tests and full reporting of outcomes
☐	☒	Estimates of effect sizes (e.g. Cohen's d, Pearson's r), indicating how they were calculated

Our web collection on statistics for biologists contains articles on many of the points above.

Software and code

Policy information about availability of computer code

Data collection	NA
Data analysis	FreeSurfer version 6.0 for MRI data and R version 3.6.2. for all statistical analyses.

For manuscripts utilizing custom algorithms or software that are central to the research but not yet described in published literature, software must be made available to editors and reviewers. We strongly encourage code deposition in a community repository (e.g. GitHub). See the Nature Portfolio guidelines for submitting code & software for further information.

Data

Policy information about availability of data

All manuscripts must include a data availability statement. This statement should provide the following information, where applicable:

- Accession codes, unique identifiers, or web links for publicly available datasets
- A description of any restrictions on data availability
- For clinical datasets or third party data, please ensure that the statement adheres to our policy

All data are available after the approval of the relevant ethical committees. Kindly contact the corresponding author for use of the dataset.

Field-specific reporting

Please select the one below that is the best fit for your research. If you are not sure, read the appropriate sections before making your selection.

☒ Life sciences ☐ Behavioural & social sciences ☐ Ecological, evolutionary & environmental sciences

For a reference copy of the document with all sections, see nature.com/documents/nr-reporting-summary-flat.pdf

Life sciences study design

All studies must disclose on these points even when the disclosure is negative.

Sample size	This is a first exploratory study and no sample size assumed before the analysis.
Data exclusions	After visual inspection, we also excluded one participant because of the failure of preprocessing, and finally, 43 images were used in this study.
Replication	NA
Randomization	NA
Blinding	NA

Reporting for specific materials, systems and methods

We require information from authors about some types of materials, experimental systems and methods used in many studies. Here, indicate whether each material, system or method listed is relevant to your study. If you are not sure if a list item applies to your research, read the appropriate section before selecting a response.

Materials & experimental systems

n/a	Involved in the study	
☒	☐	Antibodies
☒	☐	Eukaryotic cell lines
☒	☐	Palaeontology and archaeology
☒	☐	Animals and other organisms
☐	☒	Human research participants
☒	☐	Clinical data
☒	☐	Dual use research of concern

Methods

n/a	Involved in the study	
☒	☐	ChIP-seq
☒	☐	Flow cytometry
☐	☒	MRI-based neuroimaging

Human research participants

Policy information about studies involving human research participants

Population characteristics	In the IN-STEP project, we registered 53 individuals with UHR and 37 patients with FEP. Of these, 38 (71.7%) individuals with UHR and 29 (78.4%) patients with FEP who were measured using one or more modalities within 90 days of registration (including cognitive battery, MMN, structural MRI, and fNIRS brain activity) and were assessed at 12-month and/or 28-month follow-ups were included. The detailed inclusion and exclusion criteria of this study are described in the protocol paper (ref 21).
Recruitment	The participants were recruited from the outpatient and inpatient units of the University of Tokyo Hospital, University of Tokyo Health Service Center, psychiatry clinics, and internet referrals.
Ethics oversight	This study was approved by the ethics committee of the Faculty of Medicine, University of Tokyo (Approval Nos. 397, 629, 630, and 2226), and all the participants, and their caregivers if the participants were under 20 years of age, provided written informed consent to participation in the project and the required measurements following a complete explanation of the experiment.

Note that full information on the approval of the study protocol must also be provided in the manuscript.

Magnetic resonance imaging

Experimental design

Design type	Structural imaging

Design specifications	NA
Behavioral performance measures	NA

Acquisition

Imaging type(s)	structural
Field strength	3.0
Sequence & imaging parameters	Procedure 1: T1-weighted images were acquired using a 3.0-Tesla MRI scanner (Signa HDxt; GE Healthcare, Milwaukee, Wisconsin), the standard 8-channel head coil, and three-dimensional Fourier-transform fast-spoiled gradient recalled acquisition with steady state (3D-FSPGR) (repetition time = 6.80 ms, echo time = 1.94 ms, flip angle = 20°, slice thickness = 1.0 mm, field of view = 240 mm, matrix = 256 × 256, number of axial slices = 176).<br><br>Procedure 2: T1-weighted images were acquired using a 3.0-Tesla MRI scanner (MR750W; GE Healthcare, Milwaukee, Wisconsin), the standard 32-channel head coil, and 3D-FSPGR (repetition time = 8.50 ms, echo time = Min Full, flip angle = 20°, slice thickness = 1.0 mm, field of view = 240 mm, matrix = 256 × 256, number of axial slices = 176).
Area of acquisition	Brain

Diffusion MRI ☐ Used ☒ Not used

Preprocessing

Preprocessing software	FreeSurfer 6.0
Normalization	Implemented in FreeSurfer.
Normalization template	DK atlas.
Noise and artifact removal	Implemented in FreeSurfer, and after the preprocessing, ComBat harmonization was performed for reducing protocol-derived differences.
Volume censoring	NA

Statistical modeling & inference

Model type and settings	NA
Effect(s) tested	NA

Specify type of analysis: ☐ Whole brain ☐ ROI-based ☒ Both

Anatomical location(s): Based on DK atlas.

Statistic type for inference (See Eklund et al. 2016)	NA
Correction	A difference of uncorrected $p < 0.001$ was considered significant according to the exploratory MRI studies using FreeSurfer features (ref 63-65) since there was no consensus on the significance threshold set in multimodal analyses and the sample sizes were too small to tolerate Bonferroni or false discovery rate corrections.

Models & analysis

n/a	Involved in the study
☒	☐ Functional and/or effective connectivity
☒	☐ Graph analysis
☒	☐ Multivariate modeling or predictive analysis
